# A study on skin tumor classification based on dense convolutional networks with fused metadata

**DOI:** 10.3389/fonc.2022.989894

**Published:** 2022-12-16

**Authors:** Wenjun Yin, Jianhua Huang, Jianlin Chen, Yuanfa Ji

**Affiliations:** ^1^ School of Information and Communication, Guilin University Of Electronic Technology, Guilin, China; ^2^ Reproductive Endocrinology Clinic, Second Xiangya Hospital of Central South University, Changsha, China

**Keywords:** skin tumor classification, DenseNet-169 model, metadata, feature fusion, CNNs

## Abstract

Skin cancer is the most common cause of death in humans. Statistics show that competent dermatologists have a diagnostic accuracy rate of less than 80%, while inexperienced dermatologists have a diagnostic accuracy rate of less than 60%. The higher rate of misdiagnosis will cause many patients to miss the most effective treatment window, risking the patients’ life safety. However, the majority of the current study of neural network-based skin cancer diagnosis remains at the image level without patient clinical data. A deep convolutional network incorporating clinical patient metadata of skin cancer is presented to realize the classification model of skin cancer in order to further increase the accuracy of skin cancer diagnosis. There are three basic steps in the approach. First, the high-level features (edge features, color features, texture features, form features, etc.). Implied by the image were retrieved using the pre-trained DenseNet-169 model on the ImageNet dataset. Second, the MetaNet module is introduced, which uses metadata to control a certain portion of each feature channel in the DenseNet-169 network in order to produce weighted features. The MetaBlock module was added at the same time to improve the features retrieved from photos using metadata, choosing the most pertinent characteristics in accordance with the metadata data. The features of the MetaNet and MetaBlock modules were finally combined to create the MD-Net module, which was then used as input into the classifier to get the classification results for skin cancers. On the PAD-UFES-20 and ISIC 2019 datasets, the suggested methodology was assessed. The DenseNet-169 network model combined with this module, according to experimental data, obtains 81.4% in the balancing accuracy index, and its diagnostic accuracy is up between 8% and 15.6% compared to earlier efforts. Additionally, it solves the problem of actinic keratosis and poorly classified skin fibromas.

## Introduction

One of the top 10 most prevalent malignancies in the world, skin cancer is most prevalent in Caucasians, where it affects over 800,000 white people annually and causes 1% of all cancer deaths (1). By 2020, data show that more than 5 million Americans will have skin cancer screenings (2). In contrast to Western nations, China has a lower than average incidence of skin cancer, but the number of hospitalized patients is rising, with an average annual growth rate of 14.67% from 2015 to 2017 (3). According to years’ worth of statistics, basal cell carcinoma comes in second with a squamous cell carcinoma to basal cell carcinoma ratio of roughly 5~10 to 1. Squamous cell carcinoma has the greatest incidence rate in China, accounting for 80.3% of skin cancer.

With increasing skin cancer patients, the diagnosis technology is also in constant updates, such as Dermoscopy (4), dermoscopy images provide more details about the chin surface, the doctor can see deeply into the skin structure, so as to improve the diagnostic accuracy, however, the technology is mainly depends on artificial, low efficiency, and diagnosis accuracy is very dependent on the doctor’s professional level, the misdiagnosis rate is higher. For skin cancer patients, survival rates are greatly improved if they are diagnosed at an early stage. With the increase of people’s demand for medical treatment, the accumulation of medical related data is continuous. The efficient use of these data is one of the important means to support the continuous progress of medical treatment. Metadata is a kind of data describing data, which is one of the effective ways to realize the efficient use and management of massive medical data. Combining it with artificial intelligence can better promote the development of medical industries. Due to the rapid development of AI technology, deep learning models have been widely used in healthcare (5), cancer classification, disease diagnosis (6) and other fields, realizing the early detection and treatment of cancer and promoting the progress of traditional medical diagnostic technology. Therefore, machine learning and deep learning have been introduced into the clinical diagnosis of skin tumors.

The authors (7) carried out a methodical comparison of the classification of skin lesions using deep learning and traditional machine learning techniques, and came to the conclusion that deep learning is superior to traditional machine learning. Deep learning can solve this issue even if the dataset only has a small number of photos by using various improvement techniques. The majority of skin tumor diagnosis studies now in existence rely on CNN (8–10), AlexNet (11), ResNet (12), EfficientNet (13), DenseNet (14) and other neural networks to classify and diagnose skin tumors, although there are limitations to these methods. The aforementioned models always remain at the level of the image and do not take into account patient demographics, or patient data. As a result, the correlation is too far and the learnt properties have certain limitations. When making a diagnosis of skin cancer, doctors also search for and take into account features of the patient, such as age, cancer history, and anatomical region. Models that incorporated skin lesion pictures and patient demographics were proposed by Kharazmin et al. (15), Liu et al. (16), Pacheco and Krohling (17), and others. All of these efforts mix the two forms of data *via* feature concatenation, which might not account for the potential connection between metadata and visual characteristics retrieved from photos even if they all indicate promising results. Li et al. (18) Recently proposed a multiplication-based data fusion approach that use one-dimensional convolution sequences of information to extract the coefficients to support the extraction of visual features from images for classification applications. When used to classify skin cancer, this method performed better than the tandem method. The method, nevertheless, was unable to change how melanoma, the deadliest type of skin cancer, is classified. The MetaBlock approach, which employs metadata to support the data classification structure and enhances the most pertinent features retrieved from images to improve classification performance, was proposed by Andre G. C. Pacheco et al. (19). Melanoma is now better classified because of this technique. Although there is still opportunity for development in classification accuracy and other indicators, the above network is still unable to make a good identification for a few forms of skin cancer, such as basal cell carcinoma and squamous cell carcinoma, due to their high resemblance.

Due to the drawbacks of the aforementioned neural networks, this paper suggests a dense convolutional network based on the MD-Net module fused with the metadata of skin cancer clinical patients. This network corrects the drawbacks of the current convolutional neural networks and increases the precision of skin tumor diagnosis. First, we pre-trained the DenseNet model on the expansive ImageNet dataset using the transfer learning approach to get pre-training weights. The advanced characteristics buried in dermoscopy images are then extracted during fine-tuning training on the skin cancer image dataset, which increases training efficiency and saves time and energy during model training.

Second, additional screening and the extraction of more pertinent and representative features using the fusion of clinical patient metadata for skin tumors. By combining the advanced capabilities of the MetaNet (18) and MetaBlock (19) modules with those of the DenseNet-169 network, the MD-Net module was created. Reweighted features are obtained by one-dimensional convolution of metadata for the MetaNet module. By combining metadata, the MetaBlock module acquires features that are closely linked. The feature fusion is then accomplished by procedures for dimension reduction and expansion. The attention of various skin tumor-specific features can be realized through the fusion of clinical patient metadata for skin tumors. This will help screen out closely related and representative features, achieve feature enhancement, improve the model’s ability to recognize specific minority classes, and increase classification accuracy.

In order to achieve category classification, the MD-Net module’s features will finally be sent into the classifier. The network model’s Balance-Accuray (BACC) index is 81.4%, which is 8%–15. 6% greater than the relevant work accuracy and superior to the feature improvement module suggested by the existing study.

## Model construction method

### DenseNet-169 model

The DenseNet (dense convolutional network) structure, which mostly borrows from the ResNet network, was proposed by Gao et al. (20) in 2017 at the CVPR conference. DenseNet proposes a more aggressive dense connection strategy than ResNet, in which each layer is connected to the feature maps of all preceding layers and utilized as the input of the subsequent layer, which is calculated using Equation (1).


(1) $$X_{L}=H_{L}\left(\left[X_{0},\;X_{1},\;\ldots \ldots ,\;X_{L-1}\right]\right)$$

Where [*X*_0_, *X*_1_, ……, *X*_*L*−1_] s the feature map from layer 0 to layer L-1. The connectedness of DenseNet saves computational effort, improves feature propagation, stimulates feature reuse, and solves the gradient disappearance problem.

Following its introduction, DenseNet has found widespread use in image recognition thanks to its great performance (21). Following a review of the literature, this study decides to use DenseNet-169 as its foundation network by combining clinical patient metadata to create a network for classifying skin tumors.

### Metadata pre-processing

Medical picture metadata standardize the information format for patient information, case information, etc. Metadata is a type of data that describes data (22). Medical professionals can quickly comprehend the patient’s physical condition in order to provide more accurate and individualized treatment plans thanks to the proposal of medical image metadata. For researchers combining artificial intelligence with medical imaging, the information beyond medical images described by metadata helps deep learning neural networks to perform better.

Additionally, the information will be filtered according to the real needs of the use and the context of the use based on the information provided by the metadata and with reference to the associated assessment criteria. The information that best matches the needs will then be chosen.

The clinical parameters of the patient are used in this study as information to assist categorize skin cancers. Gender, age, anatomical, geography; by a boolean and other metadata are primarily included. These metadata must be translated into scalars since the features they represent are distinct and disorganized. One-hot Encoding (23) is used to digitize them as a result.

One-hot encoding, also referred to as one-bit valid encoding, is the process of encoding N states into N-bit status registers, where each state is represented by a single register bit, and only one of these bits is valid at any given moment. For instance, the unique hot encoding for the gender characteristic [“male,” “female”] is “male” (1,0): and “female” (0,1):.

### MD-Net model building

**Figure 1:**
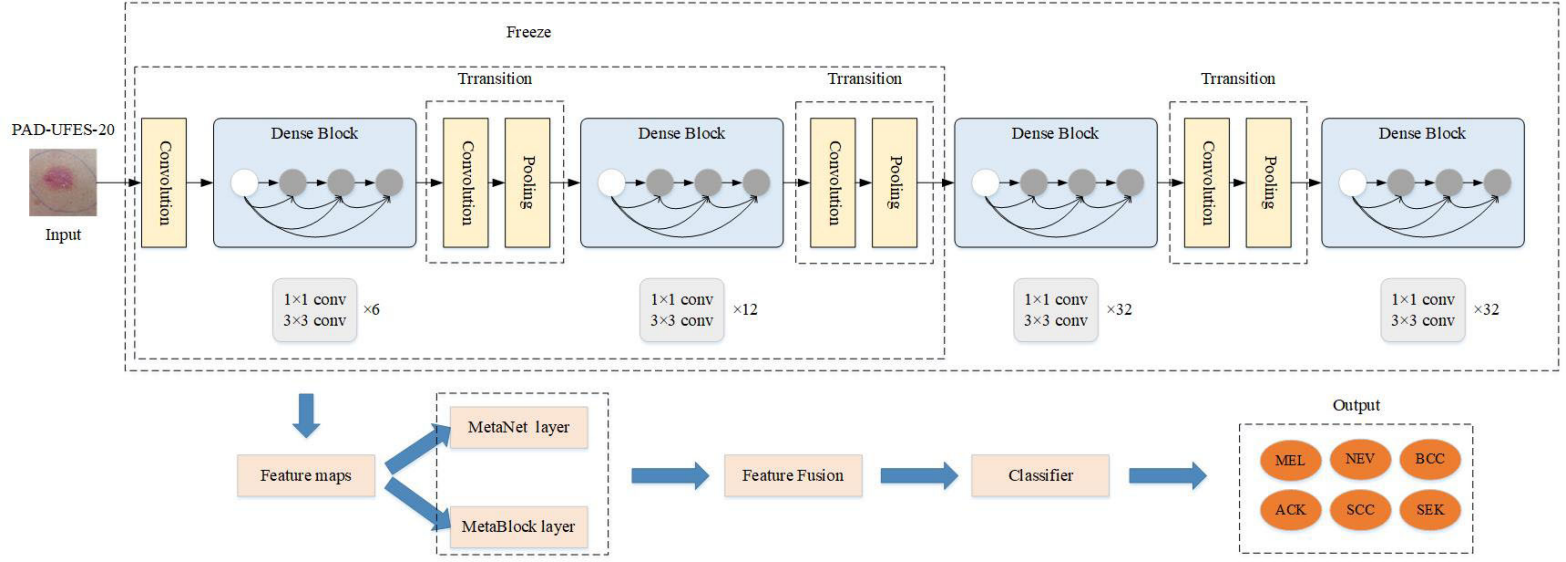
Design diagram of the improved Denset-169 network structure. The features extracted from MetaNet and MetaBlock modules are fused to construct the MD-Net module.

The skin tumor classification framework is proposed to be implemented by the MD-Net module DenseNet-169 network in this research. In **Figure 1**, the integrated network model is displayed. To begin with, the DenseNet-169 model, based on transfer learning, was presented in order to extract high-level information concealed in mirror pictures of skin cancer. Second, the hidden high-level features extracted by DenseNet-169 were separately passed through MetaNet module and MetaBlock module to obtain the weighted features and the most pertinent features between them in order to make the features extracted by the model more representative and closely related. The feature outputs of the two modules were then combined after the feature vector output of each module were shrunk and extended to have the same feature vector dimension. Finally, the input classifier produced the classification of a skin tumor.

### Transfer learning

If trained directly, the skin tumor picture dataset may not produce a satisfactory classification impact due to the tiny sample size.

Transfer learning is a machine learning technique in which the trained model parameters are used as the training parameters and the pre-training weights from big data sets are used to achieve good performance on the method’s own data set (24). In order to improve the classification accuracy of skin malignancies and reduce training time, the DenseNet-169 network is pre-trained on the ImageNet (25) dataset, and the trained weight parameters are then adjusted and trained on the target dataset.

### MetaNet module

**Figure 2:**
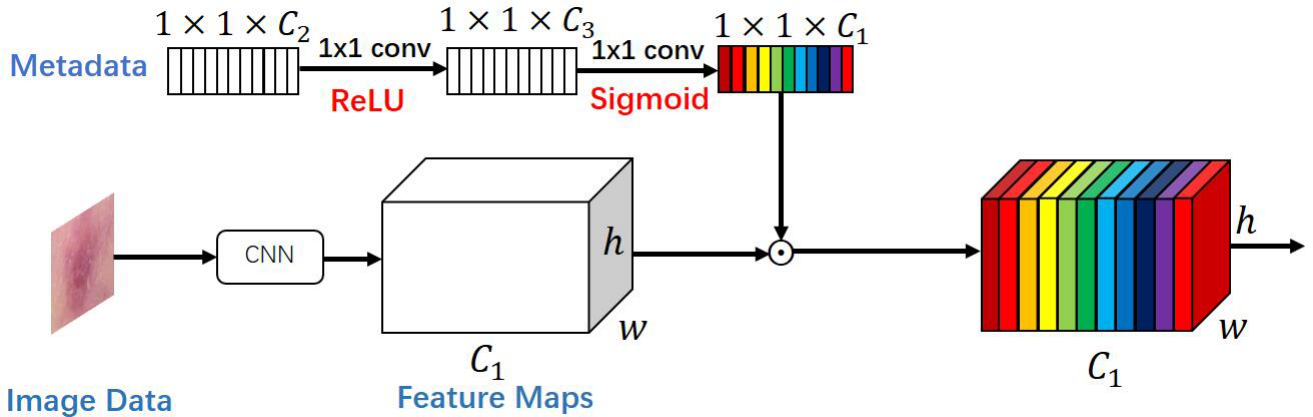
The proposed multiplication-based data fusion can make the metadata directly control the importance of each feature channel, helping the network focus on more discriminative channels.

For the categorization of visual features retrieved from photos, Li et al. (18) introduced a multiplicative data fusion approach (MetaNet), which successfully enhances the recall of skin cancer disease, especially with less training data. The schematic layout of the MetaNet module is given in **Figure 2**.

The MetaNet module is a data fusion block based on multiplication, as seen in **Figure 2**. In other words, a two-layer network of fully connected convolutional layers is first fed the metadata feature vector, with the first layer convolved followed by a modified linear unit (Relu function) and the second layer convolved followed by a Sigmoid function. The output size of this sub-network is then multiplied by the same size as the feature channel of the last convolutional layer of the feature map to obtain a re-weighted feature map. With the use of this technique, the network may concentrate on a particular area of each feature channel, increasing the precision of the skin tumor classification and allowing the metadata to interact directly with the visual features and influence the pertinent aspects of each feature channel.

### MetaBlock module

**Figure 3:**
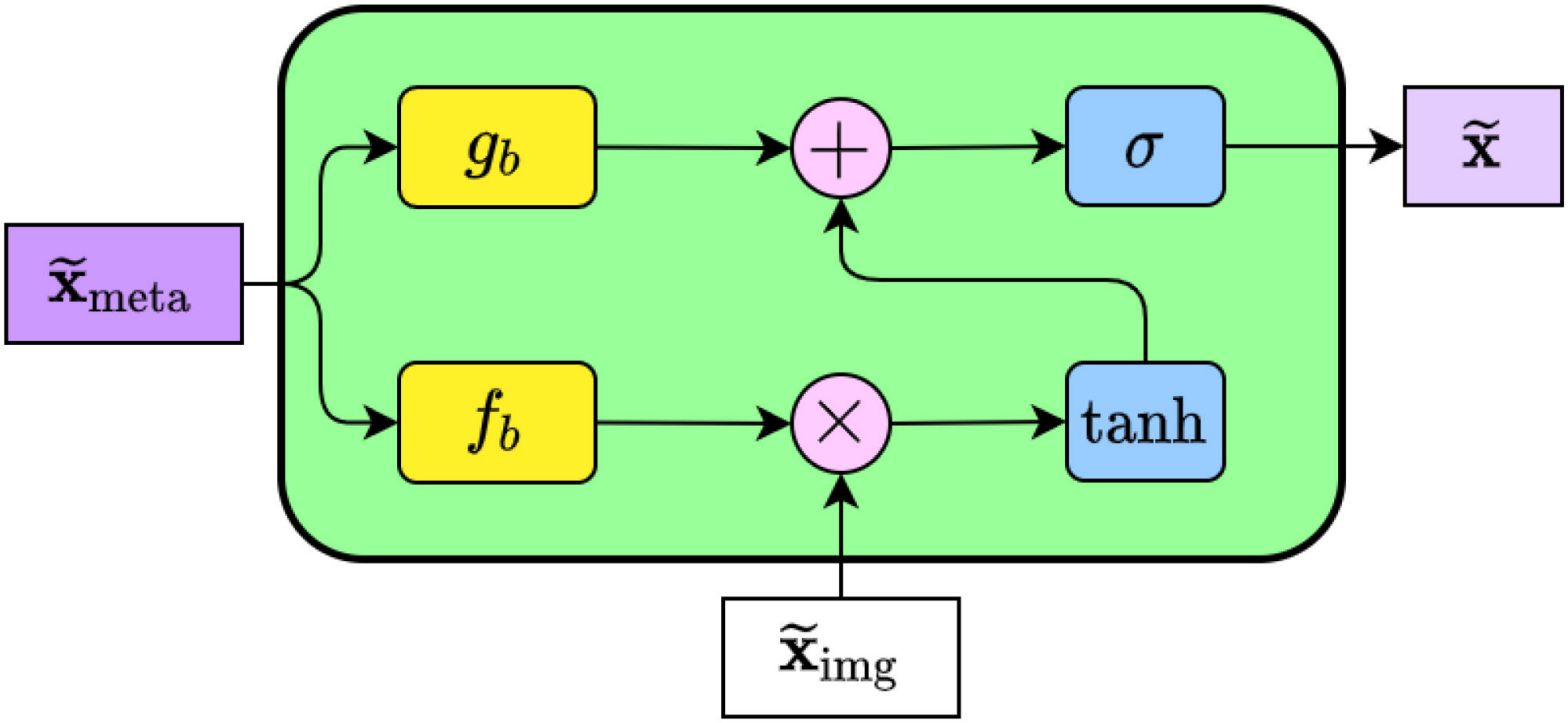
The internal structure of the MetaBlock. In summary, the block learns how to enhance the image features based on the metadata features. The output features array has the same shape as the image features.

By improving the extraction of the most pertinent features from images, i.e., directing image feature mapping based on metadata characteristics, Andre G. C. Pacheco et al. (19) Proposed the metadata processing block (MetaBlock), an attention mechanism-based approach to support skin tumor classification. The schematic diagram of the MetaBlock module is shown in **Figure 3**.

The MetaBlock module implements a batch-like normalization technique, as illustrated in **Figure 3**. This approach involves learning a function to scale and shift image features based on metadata and choosing the most important features using LSTM-like gates (26). Using equation (2).


(2) $$X=\sigma \left[\tanh\left[f_{b}\left(X_{meta}\right)⊙\leq X_{img}\right]+g_{b}\left(X_{meta}\right)\right]$$

where ⊙ is the element product, *σ*(·) And tanh (·) re the S-type (sigmoid) function, and the double tangent (hyperbolic tangent) function, respectively, Using equation (3), (4).


(3) $$f_{b}\left(X_{meta}\right)=W_{f}^{T}X_{meta}+w_{0f}$$

(4) $$g_{b}\left(X_{meta}\right)=W_{g}^{T}X_{meta}+w_{0g}$$

Where {*W*_*f*_, *W*_*g*_}∈*R* ^*d*_*meta*_×*k*_*img*_^ is the matrix weight, {*w*_0*f*_, *w*_0*g*_}∈*R* ^*k*_*img*_^ s the number of feature maps from the graph region in the image, called modifiers, by *k*_*img*_ or modifying the feature map properties and helping the model to focus on learning more important and relevant features and thus enhance the weights. After modifying the feature mapping using *k*_*img*_ the most relevant features are then filtered by hyperbolic tangent function and Sigmoid function.

According to (2), the first screening function passed, hyperbolic tangent gate, Using equation (5).


(5) $$T_{gate}=\tanh\left[f_{b}\left(X_{meta}\right)⊙X_{img}\right]$$

To raise or decrease its relevance and fulfill the screening objective, this function modifies each feature value to fall within the range (–1,1).

Then by a second screening function, the S-curve (Sigmoid gate), Using equation (6).


(6) $$S_{gate}=\sigma \left[T_{gate}+g_{b}\left(X_{meta}\right)\right]$$

This function’s goal is to output the most important feature by moving the value through the preceding gate to a range between (0,1).

Briefly stated, the MetaBlock module was designed with the intention of transmitting information to the *g*_*b*_ d *f*_*b*_ functions to produce modification coefficients. After that, the Tanh gate is used to modify the eigenvalues. The output features from the previous phase are then chosen, and the most pertinent features are output, using the Sigmoid gate.

## Experiments and results

### Dataset introduction

In this study, the proposed approach is assessed using data from two datasets of skin lesions:

PAD-UFES-20 (27): The dataset contains 2298 dermoscopic samples from six different types of skin lesions. The patient’s age, lesion location, lesion diameter, lesion location, bleeding at lesion location, and other clinical metadata were among the 22 clinical parameters that were included in each sample along with a clinical image. Basal cell cancer (BCC), squamous cell carcinoma (SCC), actinic keratosis (ACK), seborrheic keratosis (SEK), melanoma (MEL), and nevus are the six skin lesions included in the dataset (NEV). The photos in the PAD-UFES-20 collection are all high-resolution dermoscopic images of skin lesions that can show lesion details (color, texture, etc.). That is unseen to the human eye and can be used to analyze the subcutaneous structure of the skin. The data in this work is upgraded using typical image processing techniques such noise removal and picture scaling, as well as horizontal or vertical flipping, brightness, contrast, and saturation adjustments due to the dataset’s low sample size (26, 28).

**Figure 4:**
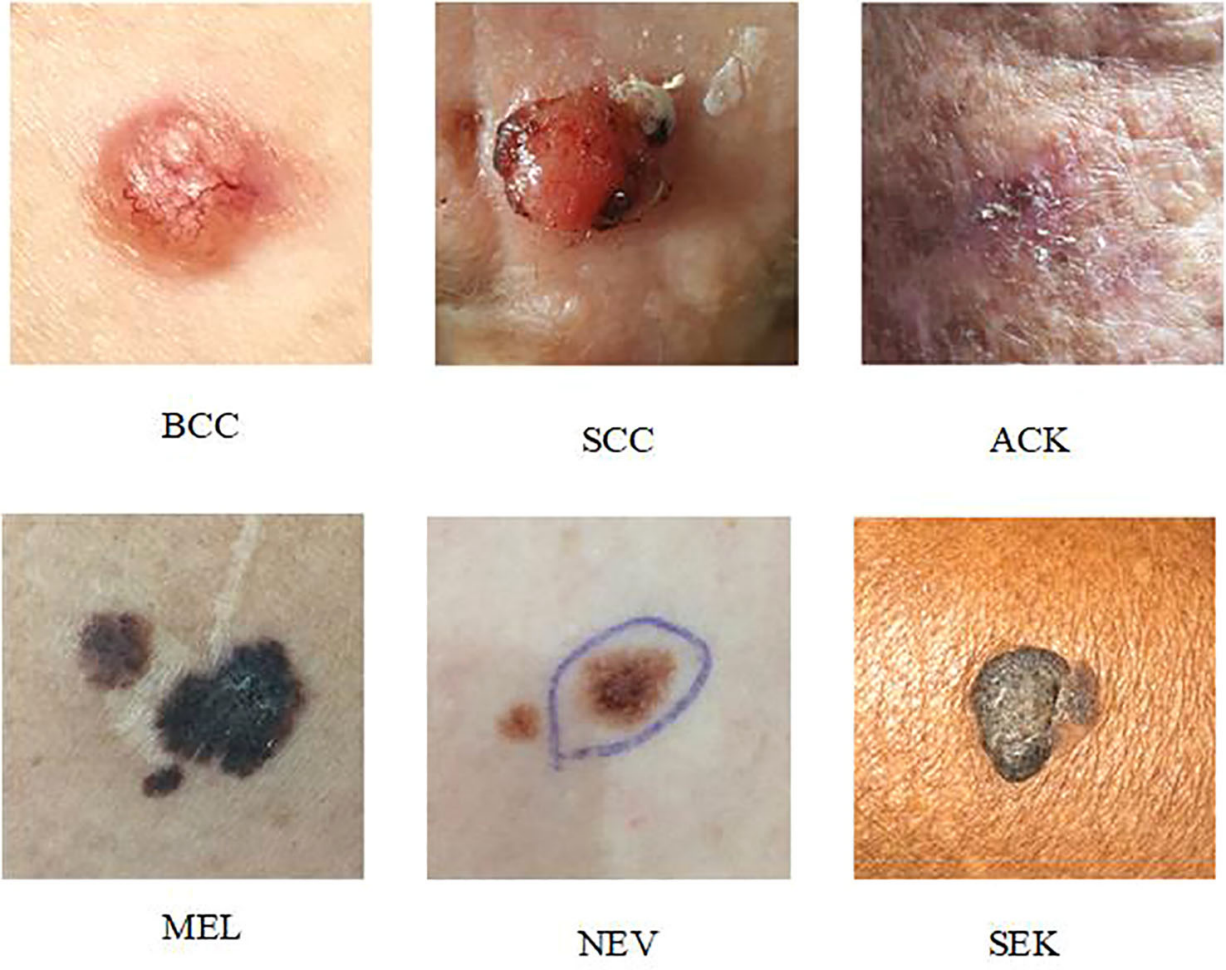
Example of the PAD-UFES-20 dataset categories.

**Figure 4** depicts an example presentation of the dataset categories.

**Table 1** displays the samples from the PAD-UFES-20 dataset for each category.

**Table 1:** Distribution of skin disease samples was analyzed by PAD-UFES-20.

Clinical Diagnosis	Number of samples
ACK	543
BCC	442
MEL	67
NEV	196
SCC	149
SEK	215

ISIC 2019 (29): This dataset includes 8238 private photos in addition to 25331 public photographs that represent eight different types of skin lesions. Age, sex, and anatomical area are the three clinical metadata that are included in each sample. The dataset includes eight skin lesions: cutaneous fibroma (DF), actinic keratosis (ACK), benign keratosis (BKL), melanin nevus (MEL), melanocytic nevus (NEV), basal cell carcinoma (BCC), and squamous cell carcinoma (SCC).

**Figure 5:**
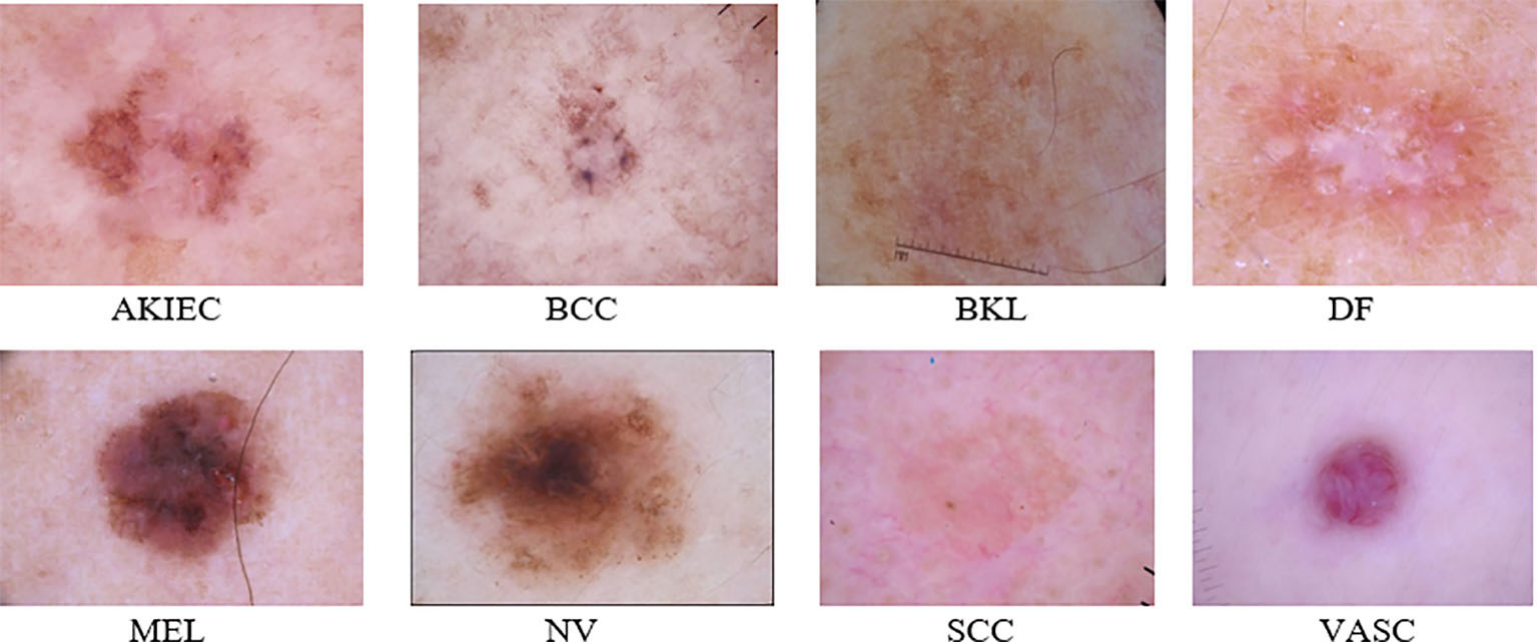
An example of the ISIC 2019 dataset categories is shown.

**Figure 5** displays an illustration of the ISIC 2019 dataset categories.

**Table 2** displays the samples from the ISIC 2019 dataset for each category.

**Table 2:** Distribution of skin disease samples in ISIC 2019 data.

Clinical Diagnosis	Number of samples
AK	869
BKL	2624
BCC	3323
DF	239
NV	12875
MEL	4522
SCC	628
NASC	253

### Experimental evaluation index

Accuracy, Sensitivity, Specificity, and Balance Accuracy (BACC) calculations were made to assess the model’s performance in this study. BACC served as the primary evaluation index because the two datasets were unbalanced. Equation 7-10 illustrates the process used to calculate the aforementioned evaluation index.


(7) $$Accuracy=\frac{TP+TN}{TP+FP+FN+TN}$$

(8) $$Sensitivity=Recall=\frac{TP}{TP+FN}$$

(9) $$Specificity=\frac{TN}{FP+TN}$$

(10) $$Balance\;accuracy=\frac{Sensitivity+Specificity}{2}$$

**Table 3** displays the pertinent configuration of the experimental setting.

**Table 3:** Environment Configuration.

Projects	Configuration
Operating System	Windows
Python version	3.6.13
Pytorch	1.10.1
Compiler Environment	Pycharm
GPU	P100
Memory	16GB

### Performance evaluation of several models

The MD-Net module compared the outputs of multiple classic CNNs networks that retrieved features, and all models employed the weight parameters learned during pre-training on the ImageNet dataset. **Tables 4**, **5** present related comparison findings.

**Table 4:** Comparing the performance of different CNNs models incorporating MD-Net modules on the PAD-UFES-20 dataset.

Backbones	ACC	BACC	AUC
ResNet-50	0.788	0.731	0.926
Vgg-13	0.627	0.666	0.920
DenseNet-121	0.757	0.737	0.946
DenseNet-169	0.796	0.814	0.956
DenseNet-201	0.768	0.798	0.953

**Table 5:** Comparing the performance of different CNNs models incorporating MD-Net modules on the ISIC 2019 dataset.

Backbones	ACC	BACC	AUC
ResNet-50	0.818	0.814	0.976
Vgg-13	0.821	0.818	0.973
DenseNet-121	0.835	0.831	0.979
DenseNet-169	0.841	0.856	0.980
DenseNet-201	0.838	0.834	0.978

According to **Tables 4**, **5**, the MD-Net model suggested in this research shows good diagnostic performance for skin malignancies and is the best in three assessment indices on two separate datasets. As can be observed, DenseNet-169 connects each layer in a pre-feedback manner to improve the transfer of features between layers. This is made possible by the unique network topology of the DenseNet-169 network, which gives the MD-Net model outstanding feature extraction ability. b) The addition of metadata gives the model the ability to learn information outside of images, extract the most important features, and disregard irrelevant information, increasing the model’s diagnostic precision.

### Comparison with experimental findings from earlier research

**Figure 6:**
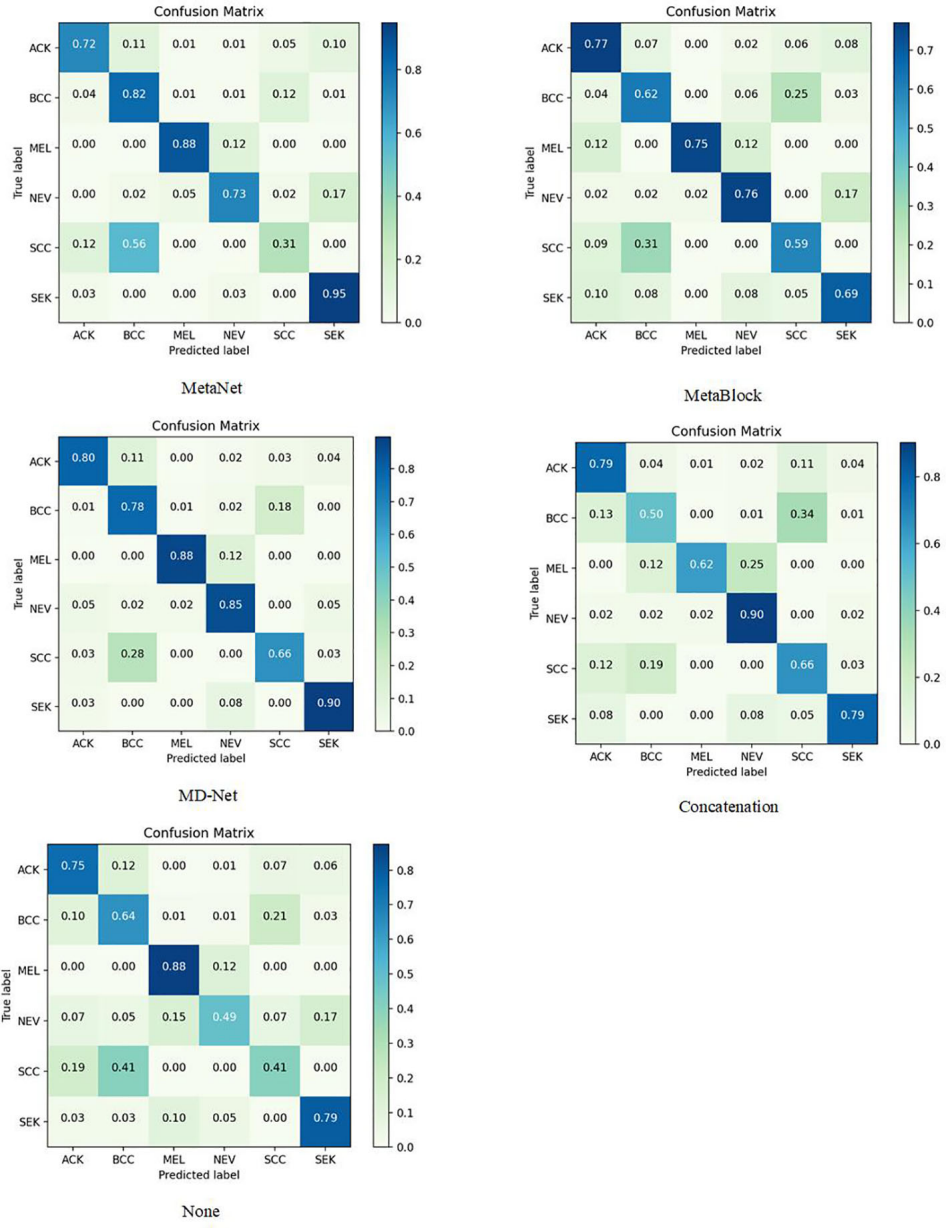
On the PAD-UFES-20 dataset, DenseNet169 combines confusion matrices of different modules.

**Figure 7:**
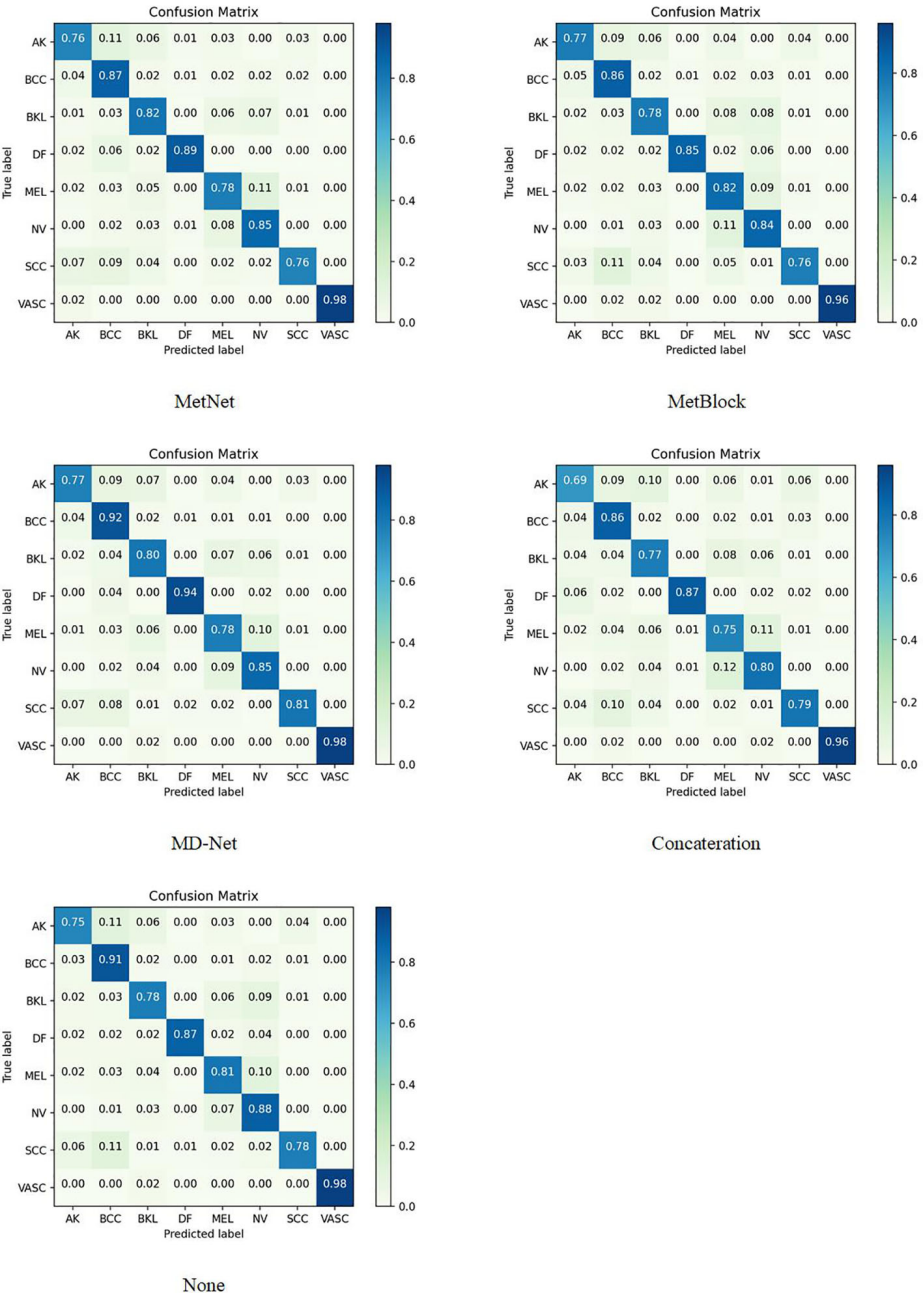
On the ISIC 2019 dataset, DenseNet169 combines the confusion matrix of different Blocks.

The sample size for model training (Batch Size) is set to 30 to avoid the overfitting issue, and the stable decreasing learning rate, Dropout, and Early Stopping are utilized to avoid data overfitting (30, 31). The parameter for “patience” is set to 10. (When the validation performance does not improve after 10 training sessions, the learning rate is reduced by half). The early stop method’s “patience” parameter was set to 15. Each model training cycle’s (Epoch’s) upper bound was set to 150. To determine how closely the actual output matches the expected output, the loss function was set to a multiclassification cross-entropy loss function and retrained on the ImageNet dataset. The two training sets chosen for evaluation in this study are as follows: The test set is 3:1 in ratio. **Tables 6**–**11** displays the classification outcomes produced by the dense convolutional network in conjunction with the MD-Net model, while **Figures 6**, **7** display the confusion matrix.

**Table 6:** Compare the performance of DenseNet-121 network fusing different modules on the PAD-UFes-20 dataset. HIGHEST AVERAGE BACC FOR EACH MODEL.

DenseNet-121			
Block	ACC	BACC	AUC
MetaNet	0.734	0.716	0.931
MetaBlock	0.713	0.702	0.930
MD-Net	0.757	**0.737**	0.946
Concatenation	0.663	0.675	0.936
None	0.715	0.678	0.916

**Table 7:** Compare the performance of DenseNet-169 network fusing different modules on the PAD-UFes-20 dataset. HIGHEST AVERAGE BACC FOR EACH MODEL.

DenseNet-169			
Block	ACC	BACC	AUC
MetaNet	0.749	0.734	0.939
MetaBlock	0.692	0.698	0.937
MD-Net	0.796	**0.814**	0.956
Concatenation	0.681	0.711	0.932
None	0.658	0.658	0.911

**Table 8:** Compare the performance of DenseNet-201 network fusing different modules on the PAD-UFes-20 dataset. HIGHEST AVERAGE BACC FOR EACH MODEL.

DenseNet-201			
Block	ACC	BACC	AUC
MetaNet	0.695	0.757	0.938
MetaBlock	0.624	0.635	0.922
MD-Net	0.768	**0.798**	0.953
Concatenation	0.721	0.726	0.932
None	0.697	0.669	0.923

**Table 9:** Compare the performance of DenseNet-121 network fusing different modules on the ISIC 2019 dataset. HIGHEST AVERAGE BACC FOR EACH MODEL.

DenseNet-121			
Block	ACC	BACC	AUC
MetaNet	0.725	0.723	0.949
MetaBlock	0.810	0.769	0.965
MD-Net	0.835	0.831	0.979
Concatenation	0.792	0.797	0.971
None	0.774	0.755	0.961

**Table 10:** Compare the performance of DenseNet-169 network fusing different modules on the ISIC 2019 dataset. HIGHEST AVERAGE BACC FOR EACH MODEL.

DenseNet-169			
Block	ACC	BACC	AUC
MetaNet	0.737	0.735	0.951
MetaBlock	0.832	0.831	0.978
MD-Net	0.841	0.856	0.980
Concatenation	0.795	0.812	0.973
None	0.784	0.776	0.968

**Table 11:** Compare the performance of DenseNet-201 network fusing different modules on the ISIC 2019 dataset. HIGHEST AVERAGE BACC FOR EACH MODEL.

DenseNet-201			
Block	ACC	BACC	AUC
MetaNet	0.831	0.828	0.975
MetaBlock	0.835	0.832	0.977
MD-Net	0.838	0.834	0.978
Concatenation	0.817	0.829	0.974
None	0.779	0.765	0.960

This section compares and analyzes the proposed MetaBlock, MetaNet, and features-concatenation modules with the MD-Net module from the study. **Tables 6**–**8** list the comparison findings for PAD datasets, while **Tables 9**–**11** list the comparison results for ISIC 2019 datasets.

The MD-Net module suggested in this research has improved in all measures, as seen by the table above. The DenseNet-169 network among them had the best experimental outcomes. The MD-Net module corrects the shortcoming that SCC and BCC are difficult to differentiate from one another due to comparable imaging features and extremely similar clinical features, hence minimizing the misclassification between them, as shown by the confusion matrix in **Figure 5**. The confusion matrix in **Figure 6** illustrates how the amount of metadata will impact classification performance, with less metadata being provided resulting in a less pronounced improvement in classification accuracy. The main distinction between this module and the previous modules is that this module adds metadata and combines features from the MetaBlock and MetaNet modules simultaneously, maximizing the utilization of features from many categories and enhancing the diagnostic precision.

According to the experimental results, the BACC index of the MD-Net module is enhanced by 8% to 15.6% in comparison to the previous study, making it more appropriate for clinical judgment. This technique is more suited for the clinical diagnosis of skin malignancies in China because it can lower the rate of SCC and BCC misdiagnosis.

## Conclusion

In order to increase the precision of skin cancer detection and streamline the process of skin tumor diagnosis, the method of skin tumor classification based on a dense convoluted network of fused information is proposed in this research. Additionally, the proposed network model’s classification outcomes on the PAD-UEFS-20 and ISIC 2019 datasets outperform those of other current networks, with strong robustness and stability. The MD-Net module suggested in this paper effectively integrates the features extracted from MetaNet module and MetaBlock module, allowing the network to pay more attention to the parts of interest, extract features with higher correlation, improve the classification accuracy of dense convolutional neural network, and subsequently assist clinicians in pre-diagnosing skin cancer tumors.

This approach also demonstrates the necessity for more clinical patient metadata, and the more clinical patient metadata provided, the greater the classification accuracy will be.

To accomplish early identification and treatment of cancer, lower the mortality rate, and also free up professionals from having to help diagnose patients, we will focus more on cancer recognition and diagnosis based on deep learning in future work. Patients should receive less unneeded care, and their discomfort should also decrease.

## Data availability statement

The datasets presented in this study are included in the article/supplementary files.

## Ethics statement

Ethical review and approval was not required for the study on human participants in accordance with the local legislation and institutional requirements. Written informed consent for participation was not required for this study in accordance with the national legislation and the institutional requirements. Written informed consent was obtained from the individual(s) for the publication of any potentially identifiable images or data included in this article.

## Author contributions

WY is mainly responsible for the writing of the paper as well as the experiments. JH is mainly responsible for the selection of the paper. JC is mainly responsible for the selection of the dataset. and YJ is mainly responsible for the correction of the paper. All authors contributed to the article and approved the submitted version.

## References

[1] Feigelson HS Powers JD Kumar M Carroll NM Pathy A Ritzwoller DP. Melanoma incidence, recurrence, and mortality in an integrated healthcare system: A retrospective cohort study. Cancer Med (2019) 8(9):4508–16. doi: 10.1002/cam4.2252 PMC6675720 31215776

[2] Siegel RL Miller KD Fuchs HE Jemal A. Cancer statistics, 2021. CA: A Cancer J Clin (2021) 71(1):7–33. doi: 10.3322/caac.21654 33433946

[3] Xu L Zhang L Tian X Zhao Y Miao Z Xue M. A study on the epidemiology of skin cancer inpatients in China. Chin J Evidence-Based Med (2020) 20(11):4. doi: 10.7507/1672-2531.202007025

[4] Havaei M Mao X Wang Y Lao Q. Conditional generation of medical images *via* disentangled adversarial inference. M Med Image Anal (2021) 72:102106. doi: 10.1016/j.media.2021.102106 34153625

[5] Xiaoqi Lu Zhicheng D Jirong W. Research on structural data extraction in surgical cases. Chin J Comput (2019) 042(12):2754–68. doi: 10.11897/SP.,j.1016.2019.02754

[6] Zhongyu Ye Menglin Wu. Choroidal neovascularization segmentation combining temporal supervision and attention mechanism. Comput Sci (2021) 48(8):118–24. doi: 10.11896/jsjkx.200600150

[7] Kassem MA Hosny KM Damaševičius R Eltoukhy MM. Machine learning and deep learning methods for skin lesion classification and diagnosis: A systematic review. Diagnostics (2021) 11:1390. doi: 10.3390/diagnostics11081390 34441324 PMC8391467

[8] Mahbod A Schaefer G Wang C Dorffner G Ecker RC Ellinger I. Transfer learning using a multi-scale and multi-network ensemble for skin lesion classification. Comput Methods Programs BioMed (2020) 193:105475. doi: 10.1016/j.cmpb.2020.105475 32268255

[9] Wang L Chen A Zhang Y Wang X Zhang Y Shen Q. AK-DL: A shallow neural network model for diagnosing actinic keratosis with better performance than deep neural networks. Diagnostics (Basel) (2020) 10(4):217. doi: 10.3390/diagnostics10040217 32294962 PMC7235884

[10] M A Sharif M Akram T Damaševičius R Maskeliūnas R. Skin lesion segmentation and multiclass classification using deep learning features and improved moth flame optimization. Diagnostics (Basel) (2021) 1(5):811. doi: 10.3390/diagnostics11050811 PMC8145295 33947117

[11] Hosny KM Kassem MA Fouad MM. Classification of skin lesions into seven classes using transfer learning with Alex net [J]. J Digital Imaging (2020) 33(5):1325–34. doi: 10.1007/s10278-020-00371-9 PMC7573031 32607904

[12] Al Masni MA Kim DH Kim TS. Multiple skin lesions diagnostics *via* integrated deep convolutional networks for segmentation and classification. Comput Methods Programs BioMed (2020) 190:105351. doi: 10.1016/j.cmpb.2020.105351 32028084

[13] Huang HW Hsu BW Lee CH Tseng VS. Development of a light-weight deep learning model for cloud applications and remote diagnosis of skin cancers. Dermatol (2021) 48(3):310–6. doi: 10.1111/1346-8138.15683 33211346

[14] Mobiny A Singh A Van Nguyen H. Risk-aware machine learning classifier for skin lesion diagnosis [J]. Clin Med (2019) 8(8):1241. doi: 10.3390/jcm8081241 PMC6723257 31426482

[15] Kharazmi P Kalia S Li H Wang Z Lee T. A feature fusion system for basal cell carcinoma detection through data-driven feature learning and patient profile. Skin Res Technol (2018) 24(2):256–64. doi: 10.1111/srt.12422 29057507

[16] Liu Y Jain A Eng C Way DH Lee K Bui P. A deep learning system for differential diagnosis of skin diseases. Nat Med (2020) 26(6):1–9. doi: 10.1038/s41591-020-0842-3 32424212

[17] Pacheco AG Krohling RA. The impact of patient clinical information on automated skin cancer detection. Comput Biol Med (2020) 116. doi: 10.1016/j.compbiomed.2019.103545 31760271

[18] Li W Zhuang J Wang R Zhang J Zheng W-S. Fusing metadata and dermoscopy images for skin disease diagnosis, in: 2020 IEEE 17th International Symposium on Biomedical Imaging (ISBI) (2020), IEEE.

[19] Pacheco AGC Krohling RA. An attention-based mechanism to combine images and metadata in deep learning models applied to skin cancer classification. IEEE J Biomed Health Inf (2021) 25(9):3554–63. doi: 10.1109/JBHI.2021.3062002 33635800

[20] Huang G Liu Z Laurens V Weinberger KQ. Densely connected convolutional networks, in: IEEE Computer Society, (2016).doi: 10.1109/CVPR.2017.243.

[21] Alhudhaif A Polat K Karaman O. Determination of COVID-19 pneumonia based on generalized convolutional neural network model from chest X-ray images. Expert Syst Appl (2021) 180:115141. doi: 10.1016/j.eswa.2021.115141 33967405 PMC8093008

[22] Gao S Jing J. The role of metadata in the organization and retrieval of web information resources. Intell Sci (2004) 22(12):1455–1,457. doi: 10.3969/j.issn.1007-7634.2004.12.013

[23] Géron A. Hands-on machine learning with scikit-learn, keras, and TensorFlow: Concepts, tools, and techniques to build intelligent systems. California, USA: O’Reilly Media (2019).

[24] Ioffe S Szegedy C. (2015). Batch normalization: Accelerating deep network training by reducing internal covariate shift, in: Proc. Int. Conf. Mach. Learn, . pp. 448–56. doi: 10.48550/arXiv.1502.03167

[25] Deng J Dong W Socher R Li L-J Li K Fei-Fei L. (2009). Imagenet: A large-scale hierarchical image database, in: Proc. IEEE Conf. Comput. Vis. Pattern Recognit, . pp. 248–55. doi: 10.1109/cvprw.2009.5206848

[26] Gessert N Nielsen M Shaikh M Werner R Schlaefer A. Skin lesion classification using ensembles of multi-resolution efficientnets with meta data. MethodsX (2020), 1-8:10.48550/arXiv.1910.03910. doi: 10.1016/j.mex.2020.100864 PMC7150512 32292713

[27] Pacheco AG Lima GR Salomo AS Krohling B. Barros L. PAD-UFES-20: A skin lesion dataset composed of patient data and clinical images collected from smartphones. Data Brief (2020) 32:1–10. doi: 10.1016/j.dib.2020.106221 PMC7479321 32939378

[28] Pacheco AG Ali A-R Trappenberg T. Skin cancer detection based on deep learning and entropy to detect outlier samples. arXiv e-prints (2019). doi: 10.48550/arXiv.1909.04525

[29] ISIC. Skin lesion analysis towards melanoma detection, in: Rdquo int. skin Imag.Collaboration (2019). Available at: https://www.isic-archive.com (Accessed Mar. 10, 2020).

[30] Prechelt L. Early stopping-but when? In: Neural networks: Tricks of the trade. Berlin: IEEE (1998).

[31] HE Y. Dual level convolutional neural network for HSI classification. Lanzhou: Lanzhou University (2020).

